# Inflammatory Myopathies and Autoimmune Gluten-related Disorders: A Scoping Review of Pathophysiological Interconnections and Hypothesis

**DOI:** 10.2174/0127722708317244240919113305

**Published:** 2024-10-01

**Authors:** Gunhild Alvik Nyborg

**Affiliations:** 1 Department of Rheumatology, Dermatology and Infectious Diseases, Oslo University Hospital, Rikshospitalet, Oslo, 0424, Norway

**Keywords:** Idiopathic inflammatory myopathies, pathophysiology, environmental risk factors, autoimmunity, gluten-related autoimmune disorders, extraintestinal manifestations

## Abstract

***Introduction*:** Anecdotal reports describe patients with concurrent idiopathic inflammatory myopathy (IIM) and celiac disease (CeD) in whom the introduction of a gluten-free diet led to dramatic improvement of myositis. We first systematically reviewed all peer-reviewed publications on concomitant IIM and duodenal biopsy-verified CeD. The collected evidence was suggestive of associations between myositis disease activity and gluten exposure in some patients with IIM-CeD.

***Methods*:** To investigate possible explanations for the observations, an exploratory review of basic pathophysiological relationships between IIM and gluten-related disorders was performed using a combined strategy of systematic and non-systematic literature searches and forward and backward citation tracking.

***Results*:** The investigations revealed close pathophysiological associations between IIM and the autoimmune gluten-related disorders CeD, dermatitis herpetiformis, and gluten ataxia. Common traits include shared genetic predisposition through HLA-DQ2.5/-DQ8, disease activity-associated autoantibodies, histopathological parallels with inflammatory cell infiltrates, and similarly distributed structural homologous transglutaminases (TGs). HLA-DQ2.5-restricted gluten-specific CD4+ T cells of a rare, uniform phenotype are reported in CeD and connective tissue disease. Expanded T-cell clones with identical phenotypes and CDR3β motifs indicate the presence of a continuous, antigen-driven T-cell response.

***Conclusion*:** The investigations revealed that the main components involved in the adaptive immune response in the CeD gut may be present in HLA-DQ2.5+/-DQ8+ IIM muscle. The collected evidence supports the notion that in some genetically predisposed patients with IIM, gluten may act as an exogenous antigen driving myositis.

***Further Research/Clinical Implications*:** To test the above hypothesis, clinical trials combined with immunological studies are needed. Meanwhile, the inclusion of HLA-DQ typing may be justified, and subsequent small-intestinal biopsies in HLA-DQ2.5/8+ individuals with IIM.

## 1 INTRODUCTION

In 1975, after it had been suggested that circulating immune complexes originating from the small intestine can lead to deposition in other organs and cause disease there [1], Henriksson *et al*. [2] published a case report describing concomitant idiopathic inflammatory myopathy (IIM) and celiac disease (CeD). Gluten withdrawal seemed to have promising effects not only on bowel symptoms but also on muscular symptoms. The authors suspected that the myositis was secondary to the enteropathy. In the following decades, several similar case reports were published, some describing a striking recovery of myositis symptoms after the initiation of a gluten-free diet (GFD).

However, few studies have been initiated to supplement these observations. In 2007, Hadjivassiliou *et al*. [3] reported 13 patients with gluten-sensitive myopathy identified at their gluten sensitivity/neurology clinic over 12 years; of these, seven were diagnosed with IIM based on muscle biopsy findings. The same year, Selva-O’Callaghan *et al*. [4] found a prevalence of CeD in IIM of 6% among 51 consecutive cases of adult IIM, and in 2017, Danielsson *et al*. [5] found a prevalence of CeD of 4.5% after screening 88 patients with IIM. Based on these findings, the prevalence of IIM-CeD appears to be higher than the estimated pooled prevalence of CeD of 1-2% [6]. The authors suggested that in some patients with IIM, particularly those with sporadic inclusion body myositis (IBM), the myopathy may be a clinical expression of gluten sensitivity. However, the pathophysiology of gluten sensitivity in IIM has not been investigated. Removing gluten from the diet is currently being discussed in patient fora. Nevertheless, the clinical associations between CeD and IIM are largely unrecognized in the medical society; no clinical guidelines exist, and doctors lack evidence to provide dietary advice. Hence, patients with a large recovery potential may go undetected for years and suffer from the burden of lung failure, muscle wasting, pain, inactivity, and potentially, early death.

Furthermore, to enhance the field in and upward direction, we systematically reviewed all peer-reviewed case reports describing patients with IIM and concomitant (preceding or succeeding) duodenal biopsy-verified CeD published between 1975 and 2020 [7]. We found 30 cases in total. The study reports [7] for a detailed description of the search methods used, PRISMA chart, and tables presenting data provided for each case, including initial symptoms and timeline, biopsy and serology results, treatment and outcome, and a discussion of the collected clinical evidence base. Briefly, in the 30 cases:

- Myositis symptoms began upon exposure to gluten in the diet in all cases except one.
- One in three reported no gastrointestinal symptoms preceding CeD diagnosis.
- Myositis disease activity decreased upon the introduction of a GFD in 14 out of 24 cases (58%), including three patients with IBM otherwise considered therapy-resistant.
- Of the 24 patients, 29% could terminate immunosuppressive medication and remained in full long-term remission with a GFD as the sole therapy.
- Only four patients experienced deterioration of myositis while on a GFD. Control duodenal biopsies were performed in two of these cases; both indicated non-adherence or refractory CeD.
- In three cases, a patient-initiated gluten rechallenge was performed. All three patients experienced acute severe myopathy accompanied by an increase in serum creatine kinase levels, and one patient was hospitalized. All three patients went into remission upon reintroduction of a GFD.

Conceivably, successful treatment of one autoimmune disease may positively impact a concomitant autoimmune disease. However, the collective evidence points towards an association between exposure to dietary gluten and myositis disease activity [7].

## 2 METHODS

This study aimed for the first time to collect and review the existing evidence base for potential pathophysiological relationships between IIM and any of the gluten-related disorders (GRDs), and such relationships were reported to propel further research in the field.

This review was performed according to the principles of the PRISMA Extension for Scoping Reviews (PRISMA-ScR) [8]. In addition, due to the nature of this work, not all checklist items were applicable.

Upon performing the clinical systematic review [7] we found that the number of articles published on gluten sensitivity in the rare, complex disorder IIM is limited. As this was the first study to directly address the research question of pathophysiological relationships between IIM and GRDs, a novel field of research into rare conditions, the aim of the literature searches pertaining to this question was to identify all relevant primary studies. Therefore, a hybrid search strategy was used [9], integrating two systematic approaches: traditional database searches and backward and forward citation tracking. The references from the reference list in our review of case reports [7] comprised the initial set of papers. For each subtopic, initial systematic literature searches were performed in (1) Medline, using search terms such as “transglutaminases or TG2 or tTG or TG3 or TG6” and “myositis or myopathy” and (2) PubMed and EMBASE, using search terms such as “myositis OR myopathy” AND “gluten OR celiac OR celiac OR amylase trypsin inhibitor OR ATI.” Supplementary non-systematic literature searches for numerous topics and authors were performed using Google Scholar. Articles and references from previous searches provided direction for improving the search phrases throughout the process, allowing detailed investigations into a string of subtopics, as presented below.

**Fig. (1):**
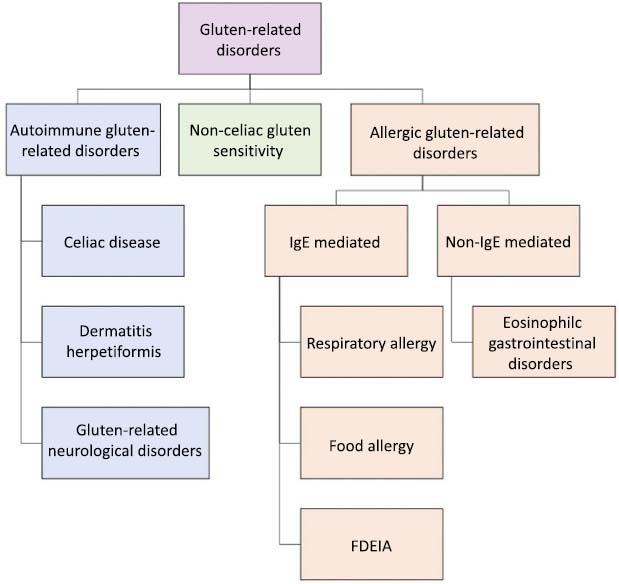
Overview of the gluten-related disorders. **Abbreviation:** FDEIA: Food-dependent exercise-induced anaphylaxis.

Although the above case reports linked IIM to CeD and to the cutaneous GRD dermatitis herpetiformis (DH), preliminary analyses spanned all categories of GRDs (Fig. **1**). No clear pathophysiological relationships were found between IIMs and allergic IgE- and non-IgE-mediated GRDs. Further, although there are unanswered questions regarding the mechanisms underlying the more recent diagnosis of non-celiac gluten sensitivity, no apparent associations with the IIMs were found. In contrast, when searching for associations between IIMs and the autoimmune GRDs CeD, DH, and gluten ataxia (GA) (Fig. **1**), strings of associations were uncovered. These findings, presented below, directed subsequent analyses.

## 3 OVERVIEW OF THE IIMS

IIMs are a group of rare systemic, relapsing-remitting autoimmune disorders belonging to the connective tissue disease (CTD) spectrum and characterized by skeletal muscle inflammation (myositis). Symptoms in other organs, such as the skin, lungs, joints, and heart, are frequent; esophageal motor abnormalities and intestinal ischemia and/or perforations occur, but gastrointestinal (GI) tract involvement is less studied [10]. IIMs were traditionally classified into dermatomyositis of adult (DM) or juvenile (JDM) onset, commonly with skin involvement, polymyositis (PM), and sporadic inclusion body myositis (IBM).

Recent insights into myositis autoantigens and myositis-specific autoantibodies (MSAs) revealed that different MSAs are associated with distinct clinical phenotypes and rarely co-occur in one patient [11]. This has led to a revised IIM classification [12, 13], with further distinction of “classical” DM; clinically amyopathic DM, often complicated by interstitial lung disease (ILD); cancer-associated DM; anti-synthetase syndrome (ASS) (PM or DM, often complicated by ILD); immune-mediated necrotizing myopathy (IMNM), sometimes related to statin use; IIM overlapping with systemic sclerosis; and IBM [11-13]. Each of these subtypes is associated with specific MSAs. The IBM subgroup displays degenerative traits and is characterized by largely therapy-resistant, progressive muscle atrophy and disability [14].

MSAs target different intracellular proteins that are ubiquitously expressed in the cytoplasm or nucleus. Pathophysiological differences related to the different MSAs may imply that different antigens may act as triggers and/or drivers in different subsets of the heterogeneous, complex IIM syndromes [11].

The IIM-CeD cases providing the clinical evidence base for this study span several decades. Therefore, MSA classification was often lacking, and IIM diagnosis was based on the traditional classification using the Bohan and Peter, Targoff, or Griggs criteria [7]. Some pathophysiological studies pertinent to this review also used the traditional classification systems, and their methodology varied according to technological advances.

## 4 OVERVIEW OF THE AUTOIMMUNE GRDS

Autoantibodies against transglutaminases (TGs) play a central role in the pathophysiology of autoimmune GRDs. TGs are a family of nine Ca^2+^-dependent enzymes that share the same amino-acid sequence (YGQCWVFA) in their catalytic sites. TGs are implicated in diverse cellular functions, such as adhesion, differentiation, proliferation, and apoptosis [15]. TG1 (keratinocyte TG), involved in the formation of strong bonds between structural proteins, is found in the stratum corneum of the epidermis alongside TG3 (epidermal TG) [16]. TG6 (neuronal TG) has been found in brain tissue, along with TG1 and TG3.

TG2 (tissue TG, tTG) is a complex, multifunctional protein involved in diverse cellular processes and diseases, including cancer, cardiovascular diseases, neurodegenerative diseases, and GRDs [17]. TG2 is largely located intracellularly in various cell lines but is also present on cell surfaces and, to a lesser degree, secreted extracellularly. Extracellular TG2 functions as a cross-linking enzyme in extracellular matrix assembly and remodeling in tissue injury and inflammation. In case of inflammation or cell death, TG2 may leak into the extracellular space and lead to locally increased extracellular TG2. Extracellular TG2 is not constitutively active *in vivo* but is transiently activated upon tissue injury and inflammation and accumulates at such sites [18].

Gluten is composed of two proteins: gliadin and glutenin. TG3 and TG6 can deamidate gluten T-cell epitopes, albeit less efficiently than TG2, and form covalent complexes with gluten peptides [19].

### 4.1 CeD

In CeD, TG2 and gluten form TG2-gluten enzyme-substrate complexes. TG2 deamidates gliadin. Deamidated gliadin peptides (DGP) can bind with high affinity to human leukocyte antigen (HLA)-DQ2.5, HLA-DQ8, or, in rare cases, HLA-DQ2.2. This creates an epitope that is effectively recognized by effector memory, gut-homing, gluten-specific CD4+ T cells not found in healthy individuals [20], that assist gluten-specific and TG2-specific B cells in antibody production. The resulting immune response in gut-associated lymphoid tissues leads to malabsorption and abdominal pain, diarrhea and/or constipation, and weight loss. Extraintestinal symptoms of fatigue, osteopathy, dental problems, and non-inflammatory myopathy are observed, but an association with myositis has not been established [21]. Diagnosis is based on serology and histological changes in small intestinal biopsies characterized by villous atrophy, crypt hyperplasia, and intraepithelial lymphocyte (IEL) infiltration [22]. Although clinical drug trials are underway, the only current treatment is a lifelong GFD. The prevalence of CeD is increased in patients with several other autoimmune diseases [21]. In CeD, as in the other autoimmune GRDs, gluten-related autoantibodies wane when a person adheres to a strict GFD, to reappear after re-exposure.

### 4.2 DH

Besides CeD, dermatitis herpetiformis, a rare skin disorder characterized by symmetrically distributed, polymorphic skin lesions, is the most studied among the autoimmune GRDs [23, 24]. TG3 is the dominant autoantigen. DH, considered an extra-intestinal manifestation of CeD, is diagnosed based on the detection of granular IgA deposits colocalizing with TG3 at the dermal-epidermal junction in perilesional skin. Most patients have mild or no GI symptoms. In approximately 75% of patients with DH, villous atrophy is observed on small intestinal biopsies; and in the remainder, an augmented presence of IEL is observed. Gluten-dependent TG2-specific IgA deposits are often found in the small intestinal mucosa [25]. CeD-associated autoantibodies are detected in a majority of patients still exposed to gluten, and more often so when villous atrophy is present. Removal of gluten from the diet has been the first-line treatment of DH since the 1970s. Healing of skin lesions may take months to years. Some patients require additional medical treatment, and this requirement has been found to correlate with poor GFD adherence.

Case reports published in the 1980s describe the development of IIM in three DH patients treated with dapsone who did not adhere to a GFD after DH diagnosis [26, 27]. Recently, another case report was published of a young man with NXP2+ amyopathic DM who was diagnosed with CeD after a small intestinal biopsy was taken during malignancy screening [28]. Serologic tests were positive for anti-TG2 autoantibodies. Five months later, after not complying with a GFD, he developed a distinct rash. A second biopsy confirmed the additional diagnosis of DH.

### 4.3 Gluten-related Neurological Disorders (GRNDs) and GA

GRNDs represent a spectrum of neurological manifestations triggered by gluten, including gluten encephalopathy, neuropathy, and GA [29]. Evidence suggests that the neurodegeneration observed in these conditions is immune-mediated [30, 31].

Gluten ataxia, one of the progressive cerebellar ataxias, generally presents with gait and lower limb ataxia of insidious onset [32]. GA is diagnosed based on serologic evidence of gluten sensitivity in patients with idiopathic, sporadic ataxia with no other known cause. Approximately one in ten patients report GI symptoms, while enteropathy with villous atrophy on small intestinal biopsies is reported in 20%-40% of cases. Anti-TG2 has been detected in approximately 40%, mostly in patients with villous atrophy. Antibodies to the autoantigen TG6 are frequently detected in GA [33]. In patients with GA who continue a gluten-containing diet, progressive disease development is observed on magnetic resonance spectroscopy of the cerebellum. Strict GFD adherence reportedly correlates with improvement or stabilization of ataxia, elimination of gluten-related antibodies, and improvement in magnetic resonance spectroscopy [34].

## 5 RESULTS

### 5.1 IIMs and Autoimmune GRDs with Common Genetic Risk Factors

Machine learning algorithms have revealed different gene expression profiles in muscle biopsies from patients with different types of myositis [35]. The dominant genetic risk factor in IIMs, including IBM, is found in the major histocompatibility complex (MHC) within alleles of the evolutionarily highly conserved multigene human 8.1 ancestral haplotype defined by alleles HLA A*0101: Cw*0701: B*0801: DRB1*0301: DQA1*0501: DQB1*0201 [36-38], which has been identified as a risk factor in several autoimmune diseases including the autoimmune GRDs. Alleles DQA1*0501 and DQB1*0201, or the HLA-DQ2.5 haplotype, are strongly associated with DRB1*0301 through linkage disequilibrium. Earlier, DRB1*0301 has been identified as the strongest risk factor in DM, JDM, and IBM. DQA1*0501 has been found to dominate in JDM [37, 39], and in a large study, 79.2% of patients with anti-synthetase syndrome and anti-Jo-1 autoantibodies carried this allele [40]. In a recent study, HLA loci associated with IIM reaching genome-wide significance was identified as HLA-DRA in IIM, HLA-DQB1 in PM, HLA-DRA in JDM, HLA-DQB1 in IBM, and HLA–DRA in Jo-1 [37]. More than 90% of patients with CeD carry HLA-DQ2.5 [41]. The majority of the remaining CeD patients carry -DQ8, encoded by HLA-DQA1*0301/0302 DQB1*0302, an additional risk factor, also in JDM. A large majority of patients with DH carry HLA-DQ2.5, and a minority carry -DQ8 [23]. GA also appears to be associated with HLA-DQ2.5 and -DQ8, albeit less closely, and evidence is still sparse [32].

A recent study of eight subgroups of IIM based on MSA/MAA status in a large international cohort revealed distinct HLA associations with autoantibody-defined subgroups [42]. Here, the GRD-associated alleles HLA-DRB1*03, HLA-DQA1*05, and HLA-DQB1*02 were overrepresented in the anti-PM/Scl and anti-Jo1/Ro52-dominated subgroups but not in the anti-U1RNP-dominated, anti-Mi2, anti-TIF1γ, or the IIM autoantibody-negative subgroup. In IMNM, DRB1*08:03, without association with autoimmune GRDs, is the strongest HLA association [43]. The strong shared HLA association indicates that antigen presentation and T-cell activation are implicated in IIM pathogenesis.

Several non-HLA loci associated with IIM have been identified [44]. TYK2, part of the JAK and antiviral pathways, is recognized as a locus associated with DM and several other autoimmune diseases, including CeD, suggesting a genetic overlap between these diseases [45].

### 5.2 Histopathological Parallels in Affected Tissues in IIMs and Autoimmune GRDs

In CeD, CD8+ and γδ+ T-cell accumulation and plasmacytosis are observed in small intestinal biopsies [20]. Subepithelial anti-TG2 IgA positivity is found along villous and crypt basement membranes and is concentrated around mucosal vessels, also in early disease stages when villous architecture is normal. IELs in the mucosa display cytotoxic properties involving perforin and granzyme B [46]. Lesions and related symptoms improve upon gluten withdrawal from the diet.

In DH, subepidermal vesicles and blisters accumulate around dermal vessels at dermal papillary tips, also during early phases [23]. Lesions are associated with lymphocyte infiltration. Granular IgA deposits accumulate at the dermal-epidermal junction. Granzyme B has been reported to be responsible for subepidermal cleavage. Autoantibodies decrease sooner than skin deposits after the introduction of a GFD.

In GA, neuropathological findings are characterized by a loss of Purkinje cells in the cerebellum and closely related areas [29]. Perivascular and intraparenchymal perforin+ granzyme B+ CD8+ T cells, thought to be cytotoxic effector cells, have been found to be particularly abundant in and around the Purkinje cell layer [47]. Marked cerebellar perivascular cuffing is observed, with infiltration of T cells, fewer B cells, and macrophages.

In IIM muscle tissue, morphological patterns distinguish different subgroups [48]. Biopsies are characterized by varying degrees of endomysial and perivascular infiltration of mononuclear cells, predominantly activated perforin+ granzyme B+ T cells and macrophages that surround, invade, and destroy muscle fibers, and of sarcolemmal and capillary complement deposits [49]. Morphological characteristics of IBM are muscle fibers invaded by CD8+ T cells and the presence of rimmed vacuoles and intracellular amyloid depositions. ASS is identified by myofiber necrosis and both hypertrophic and atrophic muscle fibers. In contrast, IMNM muscle biopsies display scattered muscle fiber necrosis, with only minimal inflammatory infiltrate.

B cells and terminally differentiated plasma cells are seen in muscle biopsies in IIM [14, 50].

DM and JDM skin is characterized by perivascular inflammation, atrophy, and microangiopathy with capillary loss, and C5b-9 membrane attack complex of the complement system in endomysial capillaries and small blood vessels in the dermis and along the dermal-epidermal junction [49].

### 5.3 MHC Class I and II Molecules and TG2 are Present in Diseased Muscle in IIM

MHC class I molecules expressed on the surface membrane of muscle fibers IIM, but not on healthy, mature muscle fibers [48, 51-56]. Sarcolemmal MHC class I expression can sometimes be seen in non-inflammatory myopathies and neurogenic disorders. Thus, the specificity of the finding is low [51]. However, the expression of MHC II on sarcolemma is a highly characteristic finding with high specificity for major subgroups of IIM, especially IBM and ASS [48, 51], and is suggested as a diagnostic tool. MHC class II molecules are notably not expressed on muscle fibers in IMNM, TIF1γ DM, or MDA5 DM [52, 53].

MHC class I/II molecules are thought to participate in IIM pathogenesis, and MHC upregulation can be an early sign in myositis. Upregulation of MHC I is found to induce myositis in a mouse model [52]. Interestingly, in cultured human myoblasts expressing HLA class I, stimulation by interferon-γ induced the expression of HLA-DR, HLA-DP, HLA-DQ, and intercellular adhesion molecule-1 (ICAM-1), after which antigen-specific proliferation was induced and the myoblasts subsequently killed by T cells [57]. In a study of AAS and DM, HLA-DR expression was found to correlate positively with the CD8+ T cell infiltrates [56]. The exact roles of these molecules in myositis pathology remain unresolved [48].

**Fig. (2):**
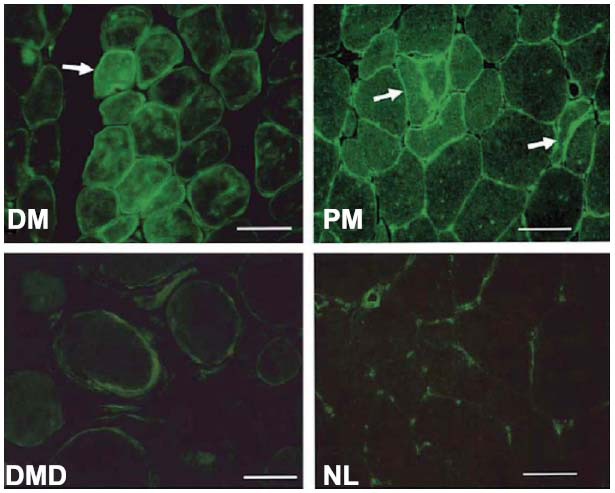
Immunohistochemical analyses of TG2 in muscle Taken from Choi *et al*, 2004 (Fig. **3**) [59]. Immunohistochemical analyses of TG2. Indirect immunofluorescence staining of TG2 was performed with frozen sections of DM, PM, DMD, and normal muscles. TG2 was strongly stained in the endomysial connective tissue and sarcolemma in IIMs. TG2 also decorates the degenerative muscle fibers in IIMs (arrow in DM indicates degenerative muscle fibers in perifascicular areas, and arrows in PM indicate non-necrotic muscle fiber and regenerative muscle fiber). **Abbreviation:** NL = Normal control. Antibody: polyclonal anti-human TGase 2, diluted 1:200, made in rabbit. License for re-use obtained from Karger Publishers.

Interestingly, increased TG2 expression and associated T-cell infiltration have been observed in muscle fibers in PM, DM, and IBM but not in healthy controls [58-60]. Choi *et al*. [59] observed strong TG2 staining in endomysial tissue and the sarcolemma in DM and PM (Fig. **2**). In Duchenne muscular dystrophy controls and healthy controls, only weak TG2 staining was observed in endomysial tissue. Except during embryonic muscle development, TG2 is normally undetectable in healthy skeletal muscle fibers [61].

**Fig. (3):**
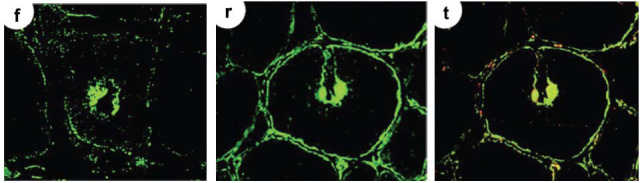
Immunohistochemical analyses reveal co*-*localization of TG2 and TG1 in IBM muscle tissue From Choi *et al*, 2000, (Extracts from Fig. (**3**), **f**, **r**, **t**) [58]. Frozen sections from muscle of IBM patient. Both anti-TG1 and anti-TG2 strongly stained vacuoles along with β-amyloid (t), and anti-TG2 staining also showed strong reaction in the endomysial connective tissues and sarcolemma (r, t). (f) Anti-TG1 strongly stained vacuoles. (r) Anti-TG2 staining showed a strong reaction in endomysial connective tissues, sarcolemma, and vacuoles. (t) Colocalization of TG1, TG2, and β-amyloid proteins. The antibodies used in the figure were anti-TG1 in (r), anti-TG2 in (r, t), anti-β-A, and double immunostaining with β-amyloid in (t). Antibodies: polyclonal anti-human TGase 1 made in goat (26) diluted 1:200; polyclonal anti-human TGase 2 made in rabbit (10) diluted 1:200; and monoclonal anti-b-amyloid precursor protein and b-amyloid peptide (Zymed Laboratories Inc., San Francisco, CA) diluted 1:10 (27). Open access under Creative Commons CC-BY license.

Choi *et al*. [58] observed increased TG2 expression in endomysial tissue, sarcolemma, and vacuoles in IBM, and TG2 and TG1 colocalized along with β-amyloid proteins in vacuolar inclusions of muscle fibers (Fig. **3**). Later, using a different specimen treatment method and, notably, IgG1 TG2 monoclonal antibodies instead of the polyclonal TG2 antibodies used by Choi *et al*., and Gendek *et al*. [60] observed intense TG2 staining in single, damaged muscle fibers and capillary endothelia in muscle biopsies only from patients with PM and DM, not healthy controls; however, they did not observe TG2 in the sarcolemma or endomysium. These discrepant results are noteworthy, also considering that in CeD, most plasma cells secrete dimeric IgA molecules. Gendek *et al*. also observed intense reaction to TG2 in damaged and regenerating muscle fibers in the perifascicular area of guinea pig muscle 72 h after the injection of sera of patients with DM, but not sera of patients with cancer or healthy controls. These results suggested the presence of a factor in the sera of patients with DM implicated in the presence of TG2 on damaged muscle fibers.

### 5.4 TG2-IgA TG2 Complexes Found in Muscle in Extraintestinal Manifestation of CeD

**Fig. (4):**
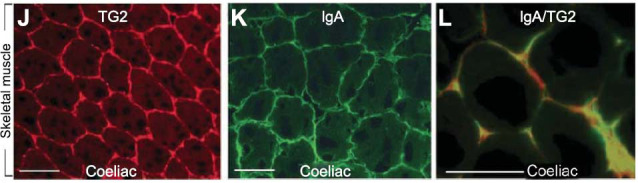
Skeletal muscle from a coeliac patient with an endomysial TG2 pattern (**J**) and endomysial IgA deposits (**K**, green). Colocalization of IgA deposits with TG2 is indicated by yellow (**L**). From Korponay-Szabo *et al* (2004), (Excerpts from Fig. (**2**), (**J-L**)) [62]. Skeletal muscle from a celiac patient with an endomysial TG2 pattern (**J**) and endomysial IgA deposits (**K**, green). Co-localization of IgA deposits with TG2 is indicated by yellow (**L**). Antibody: monoclonal mouse antibodies against TG2 (CUB7402; NeoMarkers, Fremont, California, USA). License for re-use obtained from BMJ.

Several authors have suggested that TGs are central in the pathogenesis of extraintestinal manifestations of CeD. Korponay-Szabo *et al*. [62] used double immunofluorescence to determine whether celiac TG2 IgA can reach TG2 in extraintestinal tissues. Notably, they observed colocalization of TG2-specific IgA and extracellular TG2 in skeletal muscle biopsy of a young patient with CeD not adhering to a GFD who was under assessment for neuromuscular disease because of progressive gait disturbance. In this patient, TG2 colocalized with heavy IgA deposits in endomysial structures in skeletal muscle (Fig. **4**). After the introduction of a GFD, intestinal villi normalized, and gait improved. The authors suggested that the findings indicated that the celiac disease autoantigen is widely accessible to circulating celiac autoantibodies throughout the body. Hadjivassiliou *et al*. [3] also speculated that in gluten-sensitive myositis, circulating gliadin may reach TG2 in muscle and trigger a local immune response.

In GA, granular IgA has been observed to colocalize with TG2 along small blood vessels in the perivascular areas of the cerebellum [63]. No studies of similar design in IIM patients were found.

### 5.5 Circulating CeD-associated Autoantibodies are Not Always Detected in IIM-CeD

Over the past decades, different CeD-associated antibodies have been employed for CeD screening. Tests for antibodies against native gliadin, a prolamin in wheat (AGA IgA and/or IgG), were replaced in the 1980s with tests for IgA antibodies against endomysium of smooth muscle (EMA) because of improved sensitivity and specificity [64]. In the late 1990s, TG2 (tTG), present in the endomysium of the smooth muscle of the GI tract of primates, was identified as a novel endomysial autoantigen in CeD [65]. As mentioned above, endomysial TG2 is also present in striated muscle in patients with IIM but not in healthy subjects. Following this discovery, anti-TG2 IgA replaced EMA in CeD screening, sometimes supplemented with antibodies against deamidated gliadin peptides [22].

CeD-associated antibodies are prevalent in IIM. Orbach *et al*. [66] analyzed sera from 99 IIM patients for autoantibodies related to various autoimmune diseases. They reported that IgA AGA levels were significantly elevated, and anti-TG2 levels were marginally elevated in patients with IIM compared to controls. Selva-O’Callaghan *et al*. [4] screened 51 consecutive IIM patients; AGA IgA antibodies were detected in 17 patients (31%), and all patients tested negative for anti-TG2 and EMA antibodies. Jejunal biopsies were taken in only five of the 17 AGA+ patients; of these, three were diagnosed with CeD, resulting in an IIM-CeD prevalence of 6%. In a screening of 88 IIM patients, Danielsson *et al*. [5] found 6.7% (7/88) AGA+ and 2.8% (3/88) EMA+ patients. Two of the three EMA+ patients underwent duodenal biopsy; one was diagnosed with CeD. None of the AGA+ patients underwent a biopsy. Two patients with IBM had a prior CeD diagnosis. This resulted in a total IIM-CeD prevalence of 4.5%. Notably, not all patients carrying CeD-associated antibodies were biopsied in these trials.

The cases of concomitant IIM and CeD presented in our systematic review [7] were diagnosed on the basis of muscle (IIM) and small-intestinal biopsies (CeD). As the cases spanned several decades, different CeD-associated antibodies were assessed in different patients, according to the current practice. Interestingly, 89% (16/18) were AGA+, whereas EMA was detected in 10 of 18 tested patients (56%). Anti-TG2, assessed in nine patients, was found in only two patients (22%). Anti-DGP was not tested in any of the patients. Notably, for four of the 30 patients, serology was negative, and CeD was an accidental finding in small intestinal biopsies. According to current guidelines, CeD serology screening is performed using s-anti-TG2, sometimes supplemented by s-anti-DGP [22]. Thus, screening for CeD in IIM following these guidelines may have resulted in several patients remaining undiagnosed. The presence of AGA may be more prevalent in IIM-CeD, as is observed in GA. Hadjivassiliou *et al*. [3] speculated that although the sensitivity of AGA screening in CeD is unsatisfactory, AGA may be the most sensitive marker for the spectrum of gluten-sensitive disorders. The AGA assay is currently not routinely used.

In summary, the available evidence indicates that serologic screening for CeD may be insufficient to identify gluten sensitivity in IIM. The reasons for this are unclear. Interestingly, in two s-EMA– patients with DH, anti-TG2-TG2 complexes were observed in jejunal biopsies [62]. Therefore, the absence of one of the CeD-associated autoantibodies in serum does not preclude the presence of such antibodies in organ tissues in this autoimmune GRD.

### 5.6 T-cell Characteristics in IIM *Versus* CeD

CD8+ T cells are predominant in muscle in patients with PM and IBM, whereas CD4+ T cells predominates in those with DM [67-69]. A recent T-cell transcriptomics study revealed marked differences between PM and DM in CD8+ T-cell gene expression in peripheral blood but only minor differences in CD4+ T-cell gene expression [70]. Recent research has found that myotoxicity across different IIM subsets is mediated by cells of both CD8+ and CD4+ lineages, some being highly differentiated and characterized by loss of CD28 [50, 71-73]. Expanded populations of disease-relevant CD8+ T cells have been identified in IIM muscle; some evidence suggests that these cells are clonally expanded *in situ* [67, 74]. In patients with JDM, IBM, and PM, a pattern has been found that is consistent with a limited number of antigens continuously driving the inflammatory reaction. In some patients, expanded CD8+ T-cell clones with identical phenotypes are found to persist for several years, implying a continuous, antigen-driven T-cell response [74-78]. In one patient with PM, individual cytotoxic CD8+ T cells that directly contacted, invaded, and destroyed skeletal muscle fibers expressing MHC class I molecules were found to carry a common CDR3 motif and conservative nucleotide exchanges in the CDR3 region that persisted over time [76]. However, the high variability in clonal restriction in PM and IBM and among patients suggests the presence of several local autoantigens and/or epitope spreading. In IBM, muscle-invading CD4+ and CD8+ T cells are skewed towards highly differentiated effector memory cells re-expressing CD45RA, suggesting that IBM T cells are under chronic antigen exposure [78, 79]. This study was the first to report the expansion of a differentiated CD4+ effector memory T-cell population in IIM. Among these cells were some CD28– CD4+ T cells, which may have had cytotoxic properties.

Organ damage in CeD is thought to be mediated by an increased number of cytotoxic CD8+ T cells in interplay with fewer gluten-specific CD4+ T cells [80]. Although considered crucial to CeD pathogenesis, the gluten-specific CD4+ cells make up only 1%-2% of the total CD4+ T-cell population in the intestines, and they are even less prevalent in the blood [81]. These disease-driving, gluten-specific CD4+ T cells have been extensively studied and found to have a remarkably narrow, uniform phenotype in the intestines and blood of patients with untreated CeD [82]. Notably, cells with this rare phenotype have also been identified in patients with systemic sclerosis and systemic lupus erythematosus [82], conditions belonging to the CTD spectrum along with IIM, and rheumatoid arthritis [83]. The phenotypic profile of gluten-specific CD4+ T cells changes in patients with CeD while on a GFD but rapidly reverses to that observed in patients with untreated CeD upon gluten re-exposure [80]. Clonally expanded gluten-specific CD4+ cells can persist for years in the blood and affected gut tissue and remain in the memory T-cell compartments in patients on a GFD [84]. Recently, increases in circulating γδ cells and CD8+ αβ T cells of a distinct, activated phenotype, clonally shared with tissue-resident IELs but with unknown antigen-specificity, were demonstrated in patients with CeD after gluten challenge [85]. Diverse clonotypes were found among these cells, and their frequency correlated with that of gluten-specific CD4+ T cells in the blood on day 6 of gluten challenge. Interestingly, a similar finding has been reported in DH, where increases in both circulating gluten-specific CD4+ T cells and gut-homing, activated CD8+ and γδ T cells were observed after gluten challenge [86]. These observations indicate that HLA-restricted CD4+ T cells may drive the expansion of clonally diverse αβ and γδ CD8+ T cells that may or may not be specific for the gluten antigen. There is, thus, a complex interplay between the rare gluten-specific CD4+ T cells and cytotoxic T cells in these conditions. The authors suggested that the shared γδ and CD8+ αβ T-cell clonotypes in the blood and gut tissues may be related to extraintestinal manifestations of CeD.

### 5.7 Extra-intestinal Gluten-related Autoimmunity Considered as Late-stage Complications of CeD

Although most patients do not have a prior diagnosis of CeD when they are diagnosed with DH, DH can develop over time in these patients, particularly in those not properly adhering to a GFD [87]. The frequent presence of CeD-related autoantibodies in patients with DH and of IELs in small intestinal biopsies may indicate underlying mild or latent CeD. It has been suggested that DH may represent a phenotypic change after individuals with underlying CeD have been exposed to gluten over time. The high structural similarity between TG2 and TG3 and the low prevalence of anti-TG3 in pediatric patients imply the involvement of epitope spreading and/or autoantibody cross-reactivity in the induction of TG3 autoimmunity. However, direct gluten-induced TG3 autoimmunity cannot be ruled out [88].

Similarly, the detection of anti-TG6, which is often found in GA, has been found to correlate with the duration of gluten exposure in pediatric patients with CeD [89]. GA normally develops late in life.

Among the cases included in the systematic review of IIM-CeD, 32% reported no GI symptoms, whereas 32% reported long-standing GI symptoms (6.5 years on average) before any of the two diseases were diagnosed [7]. Although speculative, some of these patients may have been unknowingly exposed to gluten as a non-tolerated antigen over time.

### 5.8 Triggers and Drivers of Autoimmunity are Not Necessarily Identical

Immune reactions leading to autoimmunity must be both initiated and maintained, and exogenous factors may act as triggers or drivers of long-term immunological reactions [90]. These factors may affect the rate of progression to clinical disease, symptoms, and clinical course of the illness.

It has been hypothesized that a viral trigger and an exogenous driver (gluten) are both required for CeD development and maintenance. Enteroviral exposure has been correlated with CeD development in genetically predisposed children after the introduction of gluten in the diet; in one study, repeated viral exposure and increased gluten intake increased the risk [91].

Results from twin studies and the fact that only a minor proportion of the phenotypic variance in IIM has been explained by genetic variants indicate the likely contribution of environmental factors [44]. Several potential environmental factors have been suggested as contributors. Viral, bacterial, and parasitic infections are suspected triggers. SARS-CoV-2 infection reportedly significantly increases IIM risk [92]. Gluten-specific CD4+ T cells with the rare phenotype found in patients with CeD and in some patients with CTD were also transiently elevated during the acute phase of influenza infection [82]. Thus, a hypothetical model can be proposed in which an infectious agent or other predicament acts as a trigger, and gluten as a driver in gluten-sensitive myositis, as suggested for CeD.

### 5.9 Potential HLA-DQ2.5/8-restricted Immune Responses Triggered by Gluten Exposure to the Skin or Lungs

Wheat and other cereals are present on surfaces and in the air in the form of fine particles. Inhalation of wheat particles is known to cause disease in individuals with wheat allergy. Whether exposure to gliadin pollution can also drive HLA-DQ2.5 or -DQ8-restricted immune responses locally in the airways and skin has not yet been elucidated. However, TG2 is present intracellularly in several airway cell lines [17]. Extracellular TG2 is known to increase in inflammatory conditions [18]. Chronic inflammation may thus lead to chronically increased extracellular TG2 levels. Some evidence suggests that in CeD, intestinal luminal TG2 stems from shed enterocytes, and shedding is increased in patients with CeD compared to healthy individuals [93]. In DH, lesions classically occur in regions of trauma where epidermal damage is thought to cause the shedding of TG3 from the spinous layer to the upper dermis, where it binds to circulating anti-TG3 IgA [24]. In patients with IIM and ILD, an increased number of T-cell clones is found in bronchial lavage fluid, suggesting that these cells expand *via* antigen-driven stimulation [94], but an antigen has not yet been identified. ILD can precede myositis symptoms [12], suggesting that the autoimmune reaction may begin in the lungs [67]. Studies on TG2 expression in ILD lungs are lacking. However, as a result of the inflammation, increased extracellular TG2 levels could be an expected finding. In idiopathic pulmonary fibrosis, extracellular TG2 expression in the lungs is increased [95, 96].

The overexpression of TG2 is also a feature of inflammatory skin disease in psoriasis [97, 98], another autoimmune disease associated with an increased risk of CeD [99]. No studies of TG2 in the skin in IIM patients were found. However, DM is a photosensitive disorder [100, 101]. Interestingly, UV radiation activates keratinocyte TG2, which is a critical mediator in UV-induced acute skin inflammation, which in turn increases extracellular TG2 levels in the skin [102].

In the hypothetical case of immune responses being initiated in multiple organs after multi-organ exposure to an environmental antigen, complex clinical manifestations could be expected, as observed in multisystem connective tissue diseases. In such cases, the elimination of the relevant antigen from only one compartment by initiating a GFD, for example, may prove insufficient for full recovery.

Intriguingly, in a recent study of phenotypic markers of disease-relevant T cells in DH, in which most cells were, as expected, positive for the gut-homing marker integrin β7, almost a third of the gluten-specific CD4+ cells in one patient were positive for the skin-homing marker CLA, and CCR4. CLA was also expressed by a large proportion of total CD8+ αβ and γδ T cells in DH patients, whereas among the activated CD103+ CD38+ T cells, only CD8+ αβ cells expressed CLA [86]. Although speculative until reproduced, these findings may indicate that in DH, activated CD8+ αβ cells, not necessarily gluten-specific, can home to the skin to exert effector functions there.

## 6 DISCUSSION

The compiled evidence reveals interconnections between inflammatory myopathies and autoimmune GRDs. Specifically,

- HLA-DQ2.5 and -DQ8 predominate in some IIM subsets. These allotypes have the unique feature of forming stable complexes with deamidated gluten peptides, which is a prerequisite for HLA-restricted gluten sensitivity.
- In some patients with IIM, and more often in HLA-DQ2.5/-DQ8-associated subsets, but not in healthy subjects, HLA class I and II molecules are expressed on the sarcolemma of myofibers.
- In patients with IIM but not in healthy subjects, extracellular TG2 expression on the sarcolemma and endomysium is increased.
- Gliadin peptides can cross the intestinal epithelium and circulate.
- Consequently, circulating gliadin peptides can encounter TG2 in muscle tissues in these patients. TG2 can effectively deamidate gliadin peptides.
- Deamidated gliadin peptides can bind with high affinity to HLA-DQ2.5 and/or -DQ8, found on the sarcolemma in patients with IIM, creating the epitope recognized by gluten-specific CD4+ cells, which are essential in gluten-dependent tissue damage in CeD.
- Gluten-specific, disease-relevant CD4+ cells of a highly specific phenotype, essential for CeD pathology, have been found in CeD and some CTDs.
- TG2 IgA can reach TG2 in extraintestinal tissues. Notably, in the case of the CeD patient with motor symptoms, where colocalization of TG2 and anti-TG2 was observed in the endomysium of skeletal muscle, the gait disturbance disappeared after the removal of gluten from the diet.

Tken together, this demonstrates that in some cases of HLA-DQ2.5+ and/or -DQ8+ IIM, the main necessary factors involved in driving the gluten-related adaptive immune response in gut tissue in CeD may also be present in muscle in patients with IIM. If this is verified in further research and these factors are found to act similarly in IIM muscle as in CeD gut, gluten may potentially act as a driver of myositis.

As shown above, an association has been reported between HLA alleles and autoantibody specificities in IIM [11, 42]. Gluten-specific, disease-relevant CD4+ T cells are HLA-DQ2.5, -DQ8, or DQ2.2-restricted. Thus, for IMNM and TIF1γ DM, subgroups that are not associated with these allotypes, and where MCH-II has not been reported on sarcolemma [43], the proposed mechanism for gluten-sensitivity is less likely. Conversely, these required factors are highly prevalent in IBM [4, 5, 7], a subgroup closely associated with HLA-DQ2.5 and with MHC-II molecules consistently detected on sarcolemma [48], and ASS, where similar findings are reported. However, the IIMs display large individual differences, and CDR3β motifs vary among patients, also within the same IIM subgroup. This suggests that gluten acts as a driver in only subsets of these patients. The available publications describe gluten-sensitivity in cases of DM, JDM, PM, and IBM diagnosed according to traditional diagnostic criteria [7]. Notably, the Bohan and Peter criteria do not differentiate between PM and IMNM.

Two explanations, alternative or supplementary to the clinical observations, should be noted. First, the successful treatment of one autoimmune disease may cause improvement in a concurrent autoimmune disease. Second, dietary change, including the introduction of a GFD, can rapidly induce large shifts in microbiota composition and diversity [103, 104]. The introduction of a GFD reduces exposure to not only gluten but also non-gluten wheat components, such as fructans (polysaccharides) and α-amylase/trypsin inhibitor proteins, which both influence the microbiota [105]. Host immune responses to the intestinal microbiota are compartmentalized to the mucosal surface, a single layer of epithelial cells separating the intestinal lumen from the underlying tissues. In CeD, microbial alterations upon the introduction of a GFD are shown to improve the integrity of this layer [106]. Further, cross-reactivity between bacterial mimics and gliadin epitopes has been observed after gluten challenge [107]. Importantly, several research groups have recently brought new insights into the role of the gut microbiome in IIM, uncovering gut dysbiosis with lower microbial diversity and distinct taxonomic composition in IIM patients *versus* healthy controls [108-112]. A unique taxonomic signature has been found in myositis patients with ILD [110] and in chronic ILD *versus* progressive ILD [108]. Lower microbial diversity and specific taxonomic compositions are also accompanied by changes in a number of metabolic pathways in serum [109-111], the levels of certain MSAs and MAAs [108, 109], and general disease activity [110]. Modification of gut dysbiosis by low-dose subcutaneous interleukin-2 has been found effective in patients with IIM [113, 114].

Therefore, microbial changes related to dietary changes, such as the introduction of a GFD, may impact the course of disease in IIM. However, these findings also demonstrate the potential significance of dietary treatment in IIM and underscore the importance of identifying underlying CeD in patients with IIM.

### 6.1 Strengths and Limitations

This is the first comprehensive review of existing pathophysiological evidence for gluten sensitivity in myositis and the first time the above novel hypothesis is presented. The literature searches conducted were numerous, extensive, and spanned diverse fields of medical research. The collected evidence provided direction for the work and hypothesis development. Evidence contrary to the final hypothesis was considered equally or more important than evidence supporting it, as it could lead to the rejection of the hypothesis. However, despite the extensive investigations, no contradictory evidence was uncovered. Instead, a string of leads to the developing hypothesis was found. In the case that the clinical observations of gluten-sensitive myositis should represent coincidences, the statistical probability of such search results would be considered low.

However, as this is a novel hypothesis, clinical trials or molecular studies have not yet been designed to specifically assess gluten sensitivity in myositis. Inflammatory myopathies are rare conditions for which many research questions remain unanswered. Therefore, the strength of the available evidence was often limited, and conclusive evidence was lacking.

## 7 IMPLICATIONS FOR CLINICAL PRACTICE AND FURTHER RESEARCH

Life-long IIMs are associated with significant morbidity and mortality, and medical treatment is challenging. In some cases included in our systematic review [8], the introduction of a GFD as the sole treatment resulted in full myositis recovery and the regaining of physical function. Others saw partial improvement. Hence, the identification of gluten sensitivity/CeD in patients with IIM may reduce the need for immunosuppressive treatment and improve quality of life, work-life, and societal participation.

CeD is not routinely assessed in patients with IIM. As mentioned above, approximately a third of the reported patients with IIM-CeD had few or no GI symptoms before CeD diagnosis [8]. CeD was sometimes an accidental finding after screening for GI cancer. Screening based on GI symptoms may thus have a low sensitivity. Low sensitivity may also apply to serological screening; CeD serology was often negative in reported cases of IIM-CeD. Consequently, the true prevalence of gluten sensitivity in patients with IIM is still unknown and likely higher than reported [4, 5], and an unknown number of these patients plausibly remain undiagnosed today.

Large population-based studies have been suggested as a tool to determine the true prevalence of CeD in patients with IIM [115]. However, our findings show that this approach will likely underestimate the true prevalence and fail to explain the different patterns of gluten sensitivity in IIM described here. Therefore, there is a need for clinical and molecular studies to determine the true prevalence and nature of gluten sensitivity and related autoantibodies in myositis. Studies analyzing TGs and associated autoantibodies in duodenal, muscle, and skin biopsies, gut permeability markers, and microbiota composition can provide additional information. Immunological studies can identify gluten-specific T cells present in the blood or diseased tissue in patients with IIM and their phenotypes. As gluten-specific CD4+ T cells comprise a minor proportion of T cells in the intestinal tissues and blood, targeted methods must be used to visualize these cells [116].

The results of this research may pave the way for new clinical guidelines to identify and assess patients with IIM who may benefit from a GFD.

## CONCLUSION

This scoping review was spurred by a number of case reports of patients suffering from idiopathic inflammatory myopathy who reportedly improved or recovered fully from their myositis upon starting a gluten-free diet after being diagnosed with celiac disease that can present without classical symptoms and serology. The investigations uncovered several pathophysiological interconnections between HLA-DQ2.5+/-DQ8+ IIM and the autoimmune gluten-related disorders of celiac disease, dermatitis herpetiformis, and gluten ataxia. Overall, the available evidence supports the notion that in some genetically predisposed HLA-DQ2.5+ and/or HLA-DQ8+ patients with IIM, gluten may act as a driver of adaptive immune responses in diseased muscle, leading to organ damage. In such cases, patients with IIM may benefit from a gluten-free diet. If later research can confirm these associations, gluten-sensitive myositis may represent an extra-intestinal manifestation of CeD and be classified as an autoimmune gluten-related disorder.

Until this hypothesis is tested and further evidence presented, CeD assessment in patients with IIM with HLA typing and subsequent duodenal biopsies for DQ2.5+ or DQ8+ patients can be justified and considered, especially in patients with IBM, also in patients with sparse gastrointestinal symptoms and negative CeD-related serology.

## AUTHORS’ CONTRIBUTIONS

The author confirms sole responsibility for the following: study conception and design, data collection, analysis and interpretation of results, and manuscript preparation.

## Data Availability

The authors confirm that the data and supportive information are available within the article.

## LIST OF ABBREVIATIONS

AGA: Anti-gliadin Antibodies
ASS: Anti-synthetase Syndrome
CeD: Celiac Disease
CTD: Connective Tissue Disease
DGP: Deamidated Gliadin Peptides
DH: Dermatitis Herpetiformis
DM: Dermatomyositis
EMA: Endomysium Antibody
GA: Gluten Ataxia
GFD: Gluten-Free Diet
GI: Gastrointestinal
GRD: Gluten-Related Disorder
HLA: Human Leukocyte Antigen
IBM: Inclusion Body Myositis
IEL: Intra-epithelial Lymphocyte
Ig: Immunoglobulin
IIM: Idiopathic Inflammatory Myopathy
IIM-CeD: Concomitant IIM and CeD
ILD: Interstitial Lung Disease
IMNM: Immune-mediated Necrotizing Myopathy
JDM: Juvenile Dermatomyositis
MAA: Myositis Associated Antibody
MHC: Major Histocompatibility Complex
MSA: Myositis Specific Antibody
PM: Polymyositis
TG: Transglutaminase

## STANDARDS OF REPORTING

PRISMA guidelines and methodology were followed.

## SUPPLEMENTARY MATERIAL

PRISMA checklist is available as supplementary material on the publisher’s website along with the published article.
